# Angiopoietin-like Proteins 4 and 8 in Diabetic Complications: Associations with Neuropathy and Metabolic Parameters in Type 2 Diabetes

**DOI:** 10.3390/jcm15103631

**Published:** 2026-05-09

**Authors:** Yuliyan Naydenov, Vera Karamfilova, Diana Nikolova, Savelia Yordanova, Zdravko Kamenov, Julieta Hristova, Antoaneta Trifonova Gateva

**Affiliations:** 1 Department of Internal Medicine, Faculty of Medicine, Medical University of Sofia, 1431 Sofia, Bulgaria; dr.ynaydenov@gmail.com (Y.N.); vkaramfilova@abv.bg (V.K.); nikolowa.diana@abv.bg (D.N.); savi_gandeva@abv.bg (S.Y.); zkamenov@hotmail.com (Z.K.); 2 Department of Clinical Laboratory, Faculty of Medicine, Medical University of Sofia, 1431 Sofia, Bulgaria; jhristova@medfac.mu-sofia.bg

**Keywords:** ANGPTL4, ANGPTL8, type 2 diabetes, cardiac autonomic neuropathy, peripheral neuropathy, lipid metabolism, hepatokine

## Abstract

**Background/Objectives**: Angiopoietin-like proteins 4 and 8 (ANGPTL4 and ANGPTL8) are key regulators of lipid metabolism and inflammatory processes, with a potential role in the pathogenesis of type 2 diabetes mellitus (T2DM) and its complications. This monocentric observational study evaluated serum levels of ANGPTL4 and ANGPTL8 in 160 participants (93 patients with T2DM and 67 controls without carbohydrate disturbances) and their associations with peripheral and cardiac autonomic neuropathy. **Methods**: This is a monocentric, cross-sectional, observational study conducted at the Endocrinology and Metabolic Disorders Clinic of Alexandrovska Hospital in Sofia, involving 160 participants and approved by the Ethics Committee of Medical University–Sofia, with all subjects providing written informed consent in accordance with the Declaration of Helsinki. The main methods included detailed clinical and anthropometric assessments, diagnosis of peripheral neuropathy via the Neuropathy Disability Score (NDS), evaluation of cardiac autonomic neuropathy using heart rate variability analysis and Ewing cardiovascular reflex tests, comprehensive laboratory investigations with fasting blood samples, measurement of serum ANGPTL4 and ANGPTL8 levels by ELISA kits, and statistical analysis performed with IBM SPSS version 25, using parametric and non-parametric tests, correlations, logistic regression, and ROC curves. **Results**: ANGPTL4 levels were significantly lower in patients with T2DM (12.6 ± 23.1 ng/mL vs. 21.5 ± 29.3 ng/mL; *p* = 0.033). In a multivariate model, higher values remained associated with lower odds of T2DM (OR per 1 SD = 0.634; *p* = 0.0424). ANGPTL8 demonstrated moderate discriminatory ability for cardiac autonomic neuropathy (AUC = 0.678; *p* = 0.007) in unadjusted analysis, but the association did not persist after covariate adjustment. ANGPTL4 showed inverse correlations with body weight, basal metabolic rate, and GGT. **Conclusions**: The results support the role of ANGPTL4 as a potential biomarker in metabolic disturbances and complications in T2DM, while ANGPTL8 remains mainly insignificant after correction for potential confounding factors.

## 1. Introduction

The global prevalence of type 2 diabetes is 11.11% (affecting 589 million adults aged 20–79 years in 2024), projected to rise to 12.96% (853 million people) by 2050 [1]. Type 2 diabetes accounts for over 96% of all diabetes cases worldwide [2]. Diabetic complications affect the majority of patients with diabetes, with a substantial global burden. The TODAY study demonstrated that the cumulative incidence of any microvascular complication reached 50% by 9 years and 80.1% by 15 years after diagnosis [3]. Specifically, kidney disease developed in 54.8%, nerve disease in 32.4%, and eye disease in 51.0% of participants by 15 years. Macrovascular complications, including coronary artery disease (8.2%), heart failure (3.3%), and stroke (2.2%), also impose a substantial burden [4]. Diabetic peripheral neuropathy (DPN) affects approximately 20–50% of people with diabetes, with prevalence varying by diabetes type, duration, and diagnostic criteria used [5]. The incidence is 8.8 cases per 1000 person-years in type 1 diabetes and 24–27 cases per 1000 person-years in type 2 diabetes. Polyol pathway activation, axoglial coupling disruption, mitochondrial dysfunction, oxidative stress, and inflammation are key interconnected mechanisms that are involved in the pathophysiology of DPN [5,6]. DPN typically presents with symmetric sensory symptoms beginning in the toes and slowly progressing proximally in a stocking-glove distribution [5,7]. Symptoms include small fiber involvement (burning pain, tingling, dysesthesia, reduced pinprick and temperature sensation), large fiber involvement (numbness, balance problems, loss of vibration and proprioception, loss of protective sensation (LOPS), reduced or absent ankle reflexes) and motor involvement (weakness, muscle atrophy). LOPS is particularly important as it predisposes individuals to unrecognized trauma and diabetic foot ulcers. The American Diabetes Association recommends screening at diagnosis for type 2 diabetes, 5 years after diagnosis for type 1 diabetes, and annually thereafter. Diagnosis includes physical examination and electrodiagnostic testing in some cases. Diabetic autonomic neuropathy (DAN) affects approximately 20% of asymptomatic individuals with diabetes and typically presents late in the disease course, often coexisting with peripheral neuropathy [8]. DAN includes cardiovascular autonomic neuropathy (CAN), gastrointestinal manifestations, genitourinary dysfunction, sudomotor dysfunction, hypoglycemia unawareness, impaired neurovascular function, and pupillary abnormalities. CAN is associated with a doubled risk of silent myocardial ischemia and mortality. Early detection through HRV testing allows for timely interventions including improved metabolic control and use of ACE inhibitors and beta-blockers, which are proven effective for patients with CAN [8,9]. CAN also accelerates other diabetic complications, including stroke, heart failure, peripheral artery disease, and Charcot joints.

ANGPTL4 is predominantly expressed in adipose tissue, liver, skeletal muscle, and heart, with secretion by adipocytes, hepatocytes, cardiomyocytes, and macrophages [10,11]. ANGPTL8 is primarily expressed in the liver and adipose tissue, with the liver being the main source of circulating ANGPTL8 [12,13]. Both proteins function as potent inhibitors of lipoprotein lipase (LPL), the key enzyme responsible for hydrolyzing triglycerides from very low-density lipoproteins and chylomicrons. ANGPTL4 is a fasting- and lipid-induced factor that mediates fasting-induced repression of LPL activity by promoting LPL unfolding, cleavage, and degradation. During fasting, exercise, and cold exposure, ANGPTL4 represses local LPL activity to ensure plasma triglycerides are specifically shuttled to tissues with high energy demands. ANGPTL4 also functions as a lipid-inducible feedback regulator of LPL-mediated lipid uptake in macrophages and cardiomyocytes [10,14]. ANGPTL8 functions as a metabolic switch by forming complexes with ANGPTL3 or ANGPTL4 to differentially regulate LPL activity. After feeding, liver-derived ANGPTL3/8 complexes act in an endocrine manner with markedly increased LPL-inhibitory activity, directing triglycerides toward adipose tissue for storage. In contrast, ANGPTL4/8 complexes formed locally in adipose tissue have decreased LPL-inhibitory activity compared to ANGPTL4 alone, enabling efficient fatty acid uptake postprandially [15,16].

No studies have yet directly measured either protein specifically in patients with diabetic peripheral neuropathy (DPN) or cardiac autonomic neuropathy (CAN), but the mechanistic evidence linking them to nerve damage is substantial and multifaceted. ANGPTL4 and ANGPTL8 contribute to diabetic peripheral and autonomic neural injury through converging mechanisms involving triglyceride dysregulation, microvascular dysfunction, neuroinflammation, and—in the case of ANGPTL8—direct neurotoxic signaling via the PirB receptor pathway. ANGPTL4 inhibits LPL by promoting its unfolding and degradation, thereby raising circulating triglycerides. The mechanistic link is direct: saturated fatty acids—whose tissue delivery is governed by LPL activity—depolarize axonal mitochondria, impair mitochondrial biogenesis and trafficking, and reduce the proportion and velocity of motile mitochondria in nerve fibers, leading to distal energy failure and the characteristic stocking-glove pattern of DPN. Elevated nonesterified fatty acids also increase superoxide production and NAD(P)H oxidase activity in Schwann cells, causing oxidative–nitrosative stress that damages peripheral nerves even in the prediabetic state [17,18,19,20]. ANGPTL4 exhibits both pro- and anti-inflammatory properties depending on tissue context and post-translational processing. The N-terminal coiled-coil domain inhibits LPL and raises triglycerides, while the C-terminal fibrinogen-like domain stimulates adipose tissue lipolysis and promotes energy expenditure. In the diabetic milieu, the net effect appears to be pro-inflammatory, with ANGPTL4 activating PKC, NF-κB, and p38 MAPK signaling—the same pathways that drive neurovascular damage in diabetic neuropathy [21,22]. ANGPTL8 is secreted by neurons in the diabetic brain, where it causes neuroinflammation and synaptic damage through its receptor PirB (paired immunoglobulin-like receptor B). In neurons, it downregulates synaptic markers (PSD-95, synaptophysin) and axonal markers (neurofilament), while in microglia, it upregulates proinflammatory cytokines (TNF-α, IL-1β, IL-6) [23].

In patients with diabetes, ANGPTL8 levels are significantly elevated and show a positive correlation with triglycerides, HOMA-IR, and BMI. ANGPTL8 is a more important determinant of plasma triglyceride levels than ANGPTL3 or ANGPTL4 in diabetic patients, with patients having high levels of both ANGPTL3 and ANGPTL8 exhibiting the worst lipid profiles. Multiple regression analysis identified high ANGPTL8 as a key determinant of fasting hypertriglyceridemia in diabetes. ANGPTL4 levels are also increased in patients with impaired glucose metabolism and hepatic impairment [24]. Loss-of-function mutations in ANGPTL4 are associated with lower plasma triglycerides, higher HDL-C, and reduced cardiovascular risk, suggesting therapeutic potential [10]. However, the role of ANGPTL8 in glucose metabolism remains controversial, with some studies suggesting it may improve glucose tolerance, while others indicate it could exacerbate glucose metabolism disorders [12].

The present study aims to evaluate serum concentrations of ANGPTL4 and ANGPTL8 in patients with T2DM and to investigate their relationship with peripheral and autonomic neuropathy.

## 2. Materials and Methods

### 2.1. Study Design

This was a monocentric, cross-sectional, observational study conducted at the Endocrinology and Metabolic Disorders Clinic of Alexandrovska Hospital, Sofia. Patients were enrolled between August 2023 and September 2025. Eligible participants were between 18 and 65 years of age, had a confirmed diagnosis of T2DM with a duration between 2 and 20 years, and were able to provide informed consent. Control subjects without carbohydrate disturbances were included if they had HbA1c < 5.7%, fasting blood glucose < 6.0 mmol/L, and plasma glucose of OGTT < 7.8 mmol/L on 2nd hour. Individuals were excluded if they had coexisting neurological diseases unrelated to diabetes, acute complications of diabetes mellitus (diabetic ketoacidosis, hyperosmolar hyperglycemic state, hyperosmolar hyperglycemic coma, hypoglycemic coma), type 1 diabetes mellitus (T1DM), diagnosed neoplasm, chronic kidney disease (CKD-eGFR < 60 mL/min/1.73 m^2^ by CKD-EPI), or heart failure (NYHA class III–IV). The study was conducted according to the guidelines of the Declaration of Helsinki and approved by the Ethics Committee of Scientific Research at Medical University–Sofia (KENIMUS) (protocol code No. 11 and date of 11 July 2023). All included patients signed informed consent. All procedures were performed in accordance with the Declaration of Helsinki.

### 2.2. Clinical and Anthropometric Assessment

Detailed medical history was obtained, including diabetes duration, current medication, comorbidities, and complications. Anthropometric measurements included height (cm), body weight (kg), body mass index (BMI, kg/m^2^), waist circumference (measured midway between the lower rib margin and iliac crest at the mid-axillary line), hip circumference (at the level of the greater trochanter), waist-to-hip ratio (WHR), and waist-to-stature ratio (WSR). Body impedance assessment was performed using TanitaMC780 MA, measuring body weight, BMI, basic metabolic rate (BMR), fat mass, fat percentage, TBW%, and visceral fat rating.

### 2.3. Diagnosis of Neuropathy

#### 2.3.1. Clinical Neuropathy Assessment

Peripheral neuropathy was assessed by the Neuropathy Disability Score (NDS) using a 10 g monofilament for tactile sensitivity, 128 Hz Rydel-Seiffer tuning fork for vibration perception, thermal discriminator for temperature sensation, and reflex hammer for ankle reflexes. Each sensory modality (vibration, temperature, and pinprick) was scored as 0 = present (normal) or 1 = absent/reduced for each foot (maximum 2 points per modality). Ankle reflexes were graded as 0 = normal, 1 = present with reinforcement, or 2 = absent, for each side. The total NDS ranged from 0 to 10 points, with higher scores indicating greater neurological impairment. In accordance with previously published thresholds, an NDS > 5 was considered indicative of clinically significant peripheral neuropathy. Higher scores indicated greater neuropathic impairment [25].

#### 2.3.2. Autonomic Neuropathy Assessment

Cardiac autonomic neuropathy was evaluated using the Cardiosys Extra system (MDE GmbH, Heidelberg, Germany) under standardized conditions (morning, fasting ≥ 12 h, no caffeine/alcohol/medications affecting the cardiovascular system, 15–20 min rest). Heart rate variability (HRV) was analyzed using time-domain (SDNN, RMSSD, pNN50, HRVi) and frequency-domain (LF, HF, LF/HF) parameters. Twelve-channel ECG with a spectral analysis (variability of heart rate, QT-interval, and QT-depression) was monitored and registered. Ewing cardiovascular reflex tests were performed and scored according to Bellavere criteria (0–2 points per test; total ≥5 points = severe autonomic neuropathy; ≥2 abnormal tests = confirmed cardiac autonomic neuropathy per Toronto Diabetic Neuropathy Expert Group).

In the frequency domain, the high-frequency component (HF, 0.15–0.40 Hz) is synchronous with respiration and primarily reflects vagal (parasympathetic) tone. The low-frequency component (LF, <0.04–0.15 Hz) is associated with changes in vasomotor tone and reflects a combination of sympathetic and vagal influences. The LF/HF ratio represents the balance between low- and high-frequency components, with an increase indicating sympathetic predominance.

Time-domain analysis included the standard deviation of all RR intervals (SDRR/SDNN), the root mean square of successive differences between adjacent RR intervals (RMSSD), the number of RR intervals differing by more than 50 ms (RR50), the percentage of successive RR intervals with a difference greater than 50 ms (pNN50), and the integral of the density of the RR interval histogram divided by its height (HRVi). Patients were stratified into three groups according to age-specific reference values for the short-term (5 min) time-domain parameters (SDNN, rMSSD, pNN50): below the 25th percentile, between the 25th and 75th percentile, and above the 75th percentile.

Cardiovascular autonomic function was further evaluated using the Ewing tests. The heart rate response to deep breathing is based on respiratory sinus arrhythmia. While a continuous ECG recording was performed, the patient breathed deeply at a rate of 6 breaths per minute (5 s of inspiration and 5 s of expiration) for a total of three cycles. The difference between the highest and lowest heart rate was calculated. In the classic Ewing criteria, a heart rate variation greater than 15 beats per minute is considered normal (0 points), 11 to 14 beats per minute is borderline (1 point), and 10 beats per minute or less is abnormal (2 points).

The heart rate response to the Valsalva maneuver was assessed by asking the patient to exhale forcefully against a resistance of 40 mmHg for 15 s. This test evaluates baroreceptor function. The increased intrathoracic pressure initially causes a transient rise in arterial blood pressure, resulting in mild bradycardia (Phase I) via baroreceptor activation. This is followed by a fall in blood pressure due to reduced venous return and stroke volume, leading to compensatory tachycardia (Phase II). Upon release of the strain (Phase III), there is a further transient drop in blood pressure accompanied by an increase in heart rate due to expansion of the pulmonary vascular bed. In Phase IV, due to the activation of baroreceptors, arterial blood pressure rises sharply above baseline levels, followed by bradycardia. The Valsalva ratio, the most important derived parameter, is calculated by dividing the longest RR interval in Phase IV by the shortest RR interval in Phase II and at the beginning of Phase III. According to classic criteria, a ratio above 1.21 is normal (0 points), a ratio between 1.11 and 1.20 is borderline (1 point), and a ratio of 1.10 or less is abnormal (2 points).

The heart rate response to postural change from the supine to the upright position (30:15 ratio) was performed simultaneously to evaluate the hemodynamic adaptation. Upon standing, blood is redistributed to the lower extremities, reducing venous return to the heart and stroke volume. A physiological compensatory response is initiated, which includes an immediate response to the sharp drop in blood pressure with a marked increase in heart rate (first 30 s), followed by an early stabilization phase (after 1–2 min) and a response to prolonged orthostasis (lasting more than 5 min). The 30:15 ratio is calculated during the initial adaptation phase (first 45 s) as the ratio between the longest RR interval (around the 30th beat) and the shortest RR interval (around the 15th beat) after standing. A value greater than 1.04 is considered normal (0 points), values from 1.01 to 1.03 (1 point) are borderline, and values of 1.00 or less are abnormal (2 points). The blood pressure response to standing was monitored by periodic measurements of arterial pressure up to the 10th minute after assuming the upright position. In classic Ewing scoring, a fall of less than 10 mmHg is normal (0 points), a fall of 11 to 29 mmHg is borderline (1 point), and a fall of 30 mmHg or more is abnormal (2 points).

The blood pressure response to sustained handgrip was performed by asking the patient to squeeze a handgrip dynamometer with the dominant hand at approximately 30% of maximal voluntary contraction for 3–5 min. Blood pressure was measured on the non-dominant arm at 1 min intervals. The rise in diastolic blood pressure results from an increase in heart rate without a significant change in peripheral vascular resistance. A rise greater than 16 mmHg is considered normal (0 points), a rise of 11 to 15 mmHg is borderline (1 point), and a rise of 10 mmHg or less is abnormal (2 points).

Patients were classified into three groups according to the total score on the Bellavere scale: 0–1 points (no autonomic neuropathy), 2–4 points (borderline results, suggesting possible mild autonomic neuropathy), 5–10 points (severe autonomic neuropathy) [26]. A score of 5 or more points indicates advanced autonomic neuropathy, as it reflects involvement of both the parasympathetic and sympathetic nervous systems. For the purpose of determining the prevalence of cardiac autonomic neuropathy (CAN), it was defined as the presence of two or more abnormal tests, according to the criteria of the Toronto Diabetic Neuropathy Expert Group [27].

### 2.4. Laboratory Investigations

Fasting venous blood samples were collected after ≥12 h overnight fast. Analyses included complete blood count, HbA1c, fasting glucose, lipid profile (total cholesterol, LDL, HDL, VLDL, triglycerides), liver enzymes (ASAT, ALAT, GGT), creatinine, eGFR, uric acid, total protein, albumin, and urine albumin-to-creatinine ratio. All tests were performed in the Central Clinical Laboratory of Alexandrovska Hospital (reference laboratory for Bulgaria). Immunochemical assays, including HbA1c and other applicable parameters, were performed using the Elecsys 2010 electrochemiluminescence immunoassay analyzer (Roche Diagnostics, Mannheim, Germany), according to the manufacturer’s instructions and routine laboratory quality control procedures. Biochemical parameters were measured using validated manufacturer-specific reagents and calibrators compatible with the laboratory analyzer systems in use. Hematological analyses were carried out on an automated hematology analyzer. The urinary albumin-to-creatinine ratio (UACR) was calculated from a spot urine sample by dividing the urine albumin concentration by the urine creatinine concentration, and it was expressed as mg/g creatinine, according to standard laboratory practice. Both urinary albumin and urinary creatinine were measured using validated automated assays.

### 2.5. Cardiovascular Risk Factors

Arterial hypertension was defined as blood pressure ≥ 140/90 mmHg and/or the use of antihypertensive treatment. Dyslipidemia was defined as total cholesterol > 5.2 mmol/L and/or HDL-cholesterol < 1.3 mmol/L in women or < 1.0 mmol/L in men and/or triglycerides > 1.7 mmol/L and/or the use of lipid-lowering treatment. Metabolic syndrome was diagnosed according to the 2009 joint criteria of the International Diabetes Federation (IDF) and the American Heart Association/National Heart, Lung, and Blood Institute (AHA/NHLBI) when at least 3 out of the following 5 risk factors were present: increased waist circumference (>80 cm in women and >94 cm in men), elevated triglycerides (≥1.7 mmol/L), reduced HDL-cholesterol (<1.3 mmol/L in women and <1.0 mmol/L in men), elevated blood pressure (≥130/85 mmHg), and elevated fasting plasma glucose (≥5.6 mmol/L) [28]. The number of fulfilled criteria was also recorded for each participant.

### 2.6. Measurement of ANGPTL4 and ANGPTL8

Serum levels of ANGPTL4 and ANGPTL8 were quantified using commercially available enzyme-linked immunosorbent assay (ELISA) kits according to the manufacturer’s protocols (Shanghai Sunred Biological Technology Co., Ltd., Shanghai, China, Cat. number 201-12-3155D and 201-12-5327A).

### 2.7. Statistical Analysis

Statistical analysis was performed using IBM SPSS Statistics version 25. Data were checked for missing values, outliers, and anomalies prior to analysis. Descriptive, variation, and graphical analyses were applied. Normality of distribution was assessed using the Kolmogorov–Smirnov and Shapiro–Wilk tests, parametric methods (ANOVA and *t*-test), and non-parametric methods (Mann–Whitney and Chi-square). Descriptive analysis was employed with presentation of frequency distributions in tabular form by study groups. Variation analysis was used for calculating measures of central tendency and dispersion; furthermore, graphical visualization of the results was performed. In cases where normal distribution was not achieved even after logarithmic transformation, non-parametric tests were used. In addition, one-way analysis of variance (ANOVA) was employed for comparing multiple independent samples, the Student’s *t*-test for comparing two independent samples, the Mann–Whitney U test for non-parametric comparison of two independent samples, and the Chi-square test and Spearman’s rank correlation coefficient were used to evaluate the strength and direction of monotonic relationships between variables. Binary and multiple logistic regression was utilized to quantify the influence of the studied factors, and receiver operating characteristic (ROC) curve analysis was used to determine the optimal cut-off values of quantitative variables for classification of specific conditions. Covariates were selected based on clinical relevance and prior literature; however, formal multicollinearity diagnostics were not performed. Optimal cut-off values were determined using Youden’s index, defined as sensitivity + specificity − 1, selecting the point that maximized the combined sensitivity and specificity. A *p*-value < 0.05 was considered statistically significant, with correction for multiple comparisons performed using the Benjamini–Hochberg false discovery rate (FDR) method.

## 3. Results

### 3.1. Participants

The study included 160 participants (mean age 55.8 ± 8.7 years): 93 patients with T2DM and 67 controls. Patients with T2DM were older and had significantly higher body weight, BMI, waist circumference, WHR, and WSR (*p* < 0.05; Table 1). Mean HbA1c in the diabetes group was 7.79 ± 1.47% and fasting glucose 7.03 ± 1.61 mmol/L.

Patients with type 2 diabetes were older and had a higher body weight, as well as higher waist circumference, WHR, and WSR (indicators of visceral adiposity) compared to participants without carbohydrate disturbances (*p* < 0.05). At the time of the study, the mean glycated hemoglobin (HbA1c) level in patients with diabetes was 7.79 ± 1.47%, while the mean fasting blood glucose was 7.03 ± 1.61 mmol/L.

Regarding antidiabetic therapy, 5.7% of patients were not receiving pharmacological treatment at the time of the study, while 33% were treated with one antidiabetic medication, 29.5% with two, 19.3% with three, 10.2% with four, and 2.3% with five medications. Metformin was used by 77.4% of patients, sulfonylureas by 36.3%, dipeptidyl peptidase-4 inhibitors by 12%, glucagon-like peptide-1 receptor agonists by 20.7%, sodium-glucose cotransporter-2 inhibitors by 25.8%, and insulin therapy by 21.7%.

Peripheral diabetic neuropathy was diagnosed in 72% (N = 67) of the patients with diabetes, diabetic nephropathy in 23.7% (N = 22), cardiac autonomic neuropathy in 65.8% (N = 48), diabetic retinopathy in 14% (N = 14), and coronary artery disease in 18.7% (N = 17). Additionally, 10.9% of the patients had a history of acute myocardial infarction, 5.5% had a history of stroke, and 5.5% had peripheral arterial disease. In unadjusted analyses, ANGPTL4 levels were significantly lower in participants with type 2 diabetes compared to those without diabetes (12.6 ± 23.1 ng/mL vs. 21.5 ± 29.3 ng/mL; *p* = 0.033).

In a multivariate logistic regression model adjusted for covariates (n = 160; 90 cases with type 2 diabetes), higher ANGPTL4 values remained associated with a lower probability of diabetes (OR per 1 SD = 0.634; 95% CI 0.408–0.985; *p* = 0.0424); however, this association did not persist after correction for multiple comparisons using the Benjamini–Hochberg false discovery rate method (FDR q = 0.2969).

### 3.2. Diabetic Neuropathy

#### 3.2.1. Peripheral Neuropathy

**Figure 1:**
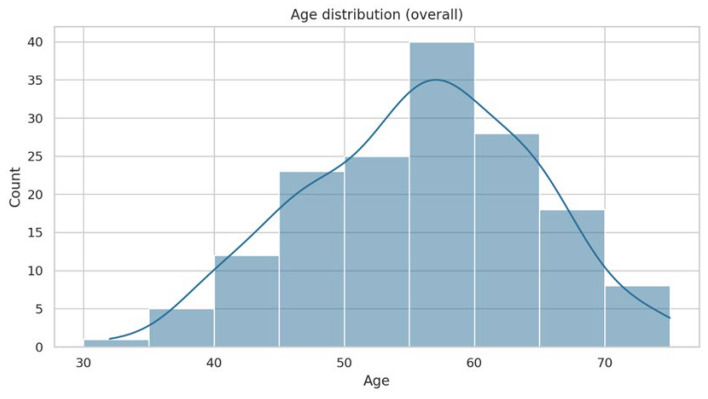
Age distribution of participants.

**Figure 2:**
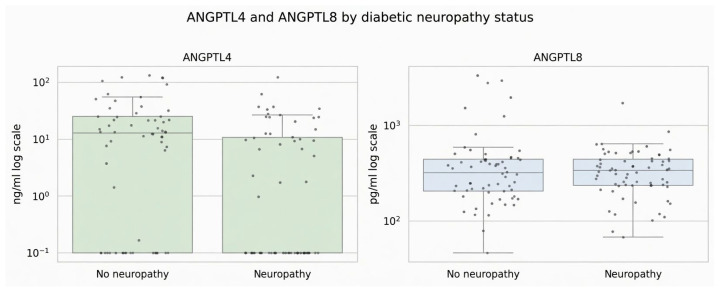
Association of ANGPTL4 and ANGPTL8 with the status of peripheral neuropathy.

ANGPTL4 was significantly lower in patients with peripheral diabetic neuropathy (9.57 ± 10.0 ng/mL vs. 23.7 ± 34.1 ng/mL, *p* = 0.001), while ANGPTL8 showed no association with the presence of peripheral neuropathy (*p* = 0.28) or with its severity as assessed by the Neuropathy Disability Score (NDS) (*p* = 0.68) (Figure 1 and Figure 2).

#### 3.2.2. Autonomic Neuropathy

Receiver operating characteristic analyses using autonomic neuropathy as the outcome showed that ANGPTL4 had a modest discriminatory ability (AUC = 0.540; *p* = 0.538), with a Youden’s index optimal cut-off of 13.73 ng/mL, yielding a sensitivity of 35.8% and specificity of 80.6% for the presence of autonomic neuropathy. In contrast, ANGPTL8 demonstrated better discrimination (AUC = 0.678; SE = 0.061; 95% CI 0.558–0.799; *p* = 0.007) (Table 2), and the Youden’s index optimal cut-off was 360.891 pg/mL, corresponding to a sensitivity of 58.5% and specificity of 77.4% for autonomic neuropathy.

**Figure 3:**
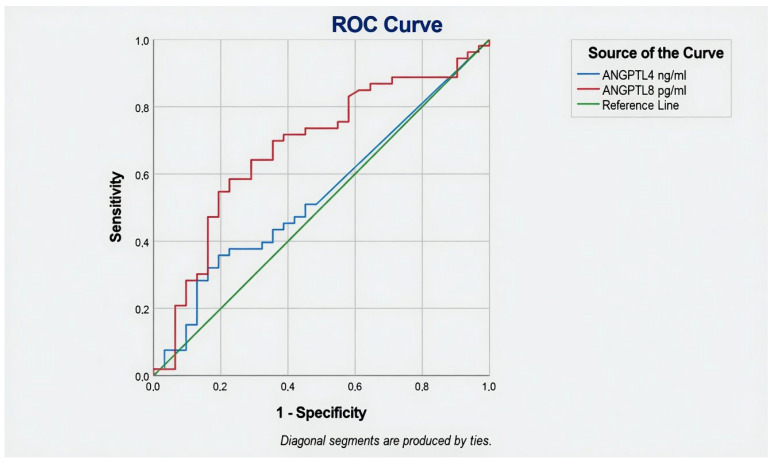
Discriminatory ability of ANGPTL4 and ANGPTL8 for the presence of cardiac autonomic neuropathy.

**Figure 4:**
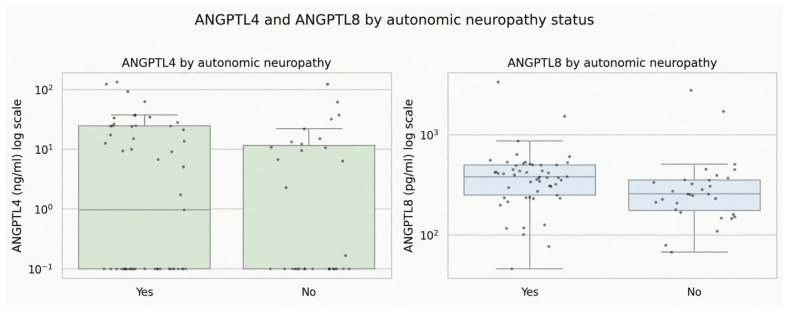
Association of ANGPTL4 and ANGPTL8 with the status of autonomic neuropathy.

In unadjusted comparisons, patients with autonomic neuropathy had significantly higher levels of ANGPTL8 (median 379.945 vs. 258.228 pg/mL; *p* = 0.0067; Cliff’s delta = 0.357) (Figure 3 and Figure 4). However, after adjustment for age, sex, BMI, HbA1c, and diabetes duration, the association did not persist. In the adjusted logistic regression model, ANGPTL8 was not significantly associated with autonomic neuropathy (OR = 1.29 per 1 SD increase in log(ANGPTL8); 95% CI 0.72–2.32; *p* = 0.387; N = 71). In contrast, ANGPTL4 showed no difference between participants with and without autonomic neuropathy in the unadjusted analysis (*p* = 0.513; N = 84), nor in the adjusted logistic regression model (OR = 0.95 per 1 SD increase in log(ANGPTL4); 95% CI 0.54–1.68; *p* = 0.864; N = 71), with the distributions in the two groups showing substantial overlap. The lower number of patients considered in the logistic regression was due to some missing data for adjusted variables.

ANGPTL4 demonstrated inverse correlations with parameters related to body size and energy expenditure. The strongest associations were observed with body weight (r = −0.224, *p* = 0.0043, FDR = 0.0231; N = 160) (Table 3) and basal metabolic rate (BMR) (r = −0.251, *p* = 0.0046, FDR = 0.0231; N = 126). A significant inverse correlation was also found with GGT (r = −0.293, *p* = 0.0011, FDR = 0.0208; N = 121). Associations with ALAT were weaker and did not remain significant after FDR correction. ANGPTL8 showed weaker and statistically non-significant correlations with these parameters. Glycemic markers (HbA1c(r = −0.044, *p* = 0.674, FDR = 0.7532 for ANGPTL4; r = 0.008, *p* = 0.943, FDR = 0.9432 for ANGPTL8; N = 92 and fasting glucose r = 0.084, *p* = 0.6111, FDR = 0.7532 for ANGPTL4; r = −0.094, *p* = 0.5712, FDR = 0.8849) did not demonstrate any substantial correlation with either ANGPTL4 or ANGPTL8 after correction for multiple comparisons. Insulin showed a weak inverse correlation with ANGPTL4, which did not remain significant after adjustment for multiple comparisons. Renal function parameters (eGFR and ACR) showed no consistent associations with either marker after FDR correction.

## 4. Discussion

The present study provides new insights into the role of circulating angiopoietin-like proteins 4 and 8 (ANGPTL4 and ANGPTL8) in patients with type 2 diabetes mellitus (T2DM) and its microvascular and macrovascular complications. In this monocentric cross-sectional cohort of 160 participants, we observed significantly lower serum ANGPTL4 concentrations in individuals with T2DM compared with non-diabetic controls, an association that remained directionally consistent in multivariate logistic regression after adjustment for key covariates, although it lost statistical significance following correction for multiple comparisons. In contrast, ANGPTL8 levels did not differ significantly between groups in adjusted models but demonstrated moderate discriminatory capacity for cardiac autonomic neuropathy (CAN) in unadjusted ROC analysis (AUC = 0.678, *p* = 0.007). Furthermore, ANGPTL4 exhibited inverse correlations with body weight, basal metabolic rate, and gamma-glutamyl transferase (GGT). However, associations with BMI and waist circumference did not reach statistical significance after correction for multiple comparisons using the Benjamini–Hochberg false discovery rate (FDR). ANGPTL8 showed weaker and largely non-significant associations with these parameters. These findings extend the current understanding of ANGPTL4 and ANGPTL8 as modulators of metabolism and inflammation, highlighting their potential differential involvement in the metabolic and neuropathic complications of T2DM [24].

In multivariate logistic regression, higher ANGPTL4 concentrations were initially associated with lower odds of T2DM, but this association lost statistical significance following FDR correction. The observation of reduced ANGPTL4 levels in T2DM raises an important point because of the emerging evidence that ANGPTL4 functions as a context-dependent regulator of lipid partitioning and insulin sensitivity [10]. ANGPTL4 is a multifunctional glycoprotein expressed in adipose tissue, liver, skeletal muscle, and heart, where it inhibits lipoprotein lipase (LPL) activity by promoting its unfolding, cleavage, and degradation, thereby directing triglycerides toward oxidative tissues during fasting states. In adipose tissue specifically, insulin (which rises after feeding) downregulates ANGPTL4 mRNA and protein in primary human adipocytes [29]. This supports the notion that ANGPTL4 is suppressed in fat tissue during feeding, allowing LPL activity to increase for lipid storage. Loss-of-function variants in ANGPTL4 are consistently associated with lower plasma triglycerides, higher HDL-cholesterol, and reduced cardiovascular risk, supporting a protective metabolic role [11,30]. In our unadjusted analyses, ANGPTL4 levels were significantly lower in participants with type 2 diabetes compared to those without diabetes, while in a multivariate logistic regression model adjusted for covariates, higher ANGPTL4 values remained associated with a lower probability of diabetes, but this association did not persist after correction for multiple comparisons. The inverse correlations observed between ANGPTL4, body weight, and basal metabolic rate further suggest that reduced ANGPTL4 may reflect or exacerbate visceral adiposity and impaired energy homeostasis. These associations remained robust after FDR correction, indicating a biologically relevant link beyond simple confounding by obesity.

A particularly intriguing finding was the significant inverse correlation between ANGPTL4 and gamma-glutamyl transferase (GGT), a liver enzyme that serves as a marker of hepatobiliary disease, oxidative stress, and cholestasis [31]. GGT is strongly associated with non-alcoholic fatty liver disease (NAFLD) and insulin resistance, both of which are highly prevalent in T2DM [32]. ANGPTL4 is upregulated in macrophages under conditions of lipid overload, where it functions as a protective feedback regulator that inhibits macrophage lipid uptake and foam cell formation [11]. In the liver, ANGPTL4 primarily functions as a local inhibitor of hepatic lipase and may be upregulated during inflammation as part of the acute phase response. The lower ANGPTL4 levels observed here, coupled with the negative correlation with GGT, may indicate a failure of this compensatory hepatic response in established T2DM, potentially contributing to the progression of hepatic steatosis and systemic inflammation. This interpretation is supported by experimental data showing that ANGPTL4 deficiency exacerbates diet-induced hepatic inflammation and fibrosis in animal models [33].

ANGPTL8 did not show independent associations with either peripheral or cardiac autonomic neuropathy after multivariable adjustment. However, the moderate AUC of 0.678 for the discrimination of CAN in unadjusted analysis is noteworthy, particularly given the high prevalence of autonomic neuropathy (65.8%) in our population. ANGPTL8 acts primarily as a postprandial metabolic switch by forming complexes with ANGPTL3 that potently inhibit LPL in oxidative tissues, thereby directing triglycerides toward adipose storage [15,24]. It is also increasingly recognized for its role in inflammatory signaling via interaction with the LILRB2/PirB receptor, which modulates macrophage recruitment and hepatic circadian rhythmicity [34]. Elevated ANGPTL8 has been linked to postprandial hypertriglyceridemia, insulin resistance, and subclinical inflammation—factors that are involved in the pathogenesis of autonomic neuropathy through microvascular endothelial dysfunction and oxidative stress [24]. The loss of statistical significance after adjustment for age, sex, BMI, HbA1c, and diabetes duration suggests that ANGPTL8 may serve as a downstream mediator rather than an independent driver of CAN. Nevertheless, the unadjusted association and the borderline elevation in patients with autonomic neuropathy warrant further investigation in larger longitudinal cohorts, especially given recent reports linking ANGPTL8 to accelerated atherosclerosis and diabetic kidney disease progression [35].

The borderline associations of ANGPTL4 with diabetic nephropathy and retinopathy in unadjusted analyses, while not surviving multiple-testing correction, are biologically plausible and consistent with the known involvement of ANGPTL proteins in microvascular pathology. ANGPTL4 has been shown to regulate vascular permeability and angiogenesis through interactions with VEGF signaling pathways, and elevated levels have previously been reported in proliferative diabetic retinopathy [36]. The direction of the association in our study (lower ANGPTL4 in patients with complications) may reflect disease-stage-dependent dynamics: compensatory upregulation in early metabolic stress followed by exhaustion in advanced microvascular damage. Similarly, the lack of robust associations for ANGPTL8 with microvascular complications after adjustment highlights the complexity of the role of these proteins, which appear to be highly context- and tissue-specific.

Several strengths of the present study enhance the reliability of our observations. The cohort was well-characterized with standardized clinical, anthropometric, and laboratory assessments, including gold-standard evaluations of both peripheral (NDS) and cardiac autonomic neuropathy (Ewing tests plus HRV analysis). Comprehensive adjustment for multiple confounders and the use of FDR correction for multiple comparisons reduced the risk of type I error. The simultaneous measurement of both ANGPTL4 and ANGPTL8 using validated ELISA methods allowed for direct comparison of their relative contributions. However, important limitations must be acknowledged, and the results should be interpreted with caution. The cross-sectional design precludes causal inference regarding whether alterations in ANGPTL levels precede or result from T2DM complications. The modest sample size (n = 160) limits statistical power, particularly for subgroup analyses of microvascular complications. Although we adjusted for key covariates, residual confounding by unmeasured factors such as diet, physical activity, or specific antidiabetic medications cannot be entirely excluded. Furthermore, circulating levels of ANGPTL proteins may not fully reflect tissue-specific expression or local paracrine actions, which could be more relevant to neuropathy pathogenesis. Finally, information on lipid-modifying medications (particularly statins and fibrates) was not available. Fibrates are potent PPARα agonists that paradoxically increase ANGPTL4 levels dramatically because ANGPTL4 is a direct PPAR transcriptional target, yet this elevation does not mediate their triglyceride-lowering effect, which occurs through separate PPARα-dependent mechanisms such as increased fatty acid oxidation and reduced VLDL secretion. Statins, by contrast, do not affect ANGPTL4 levels since they inhibit HMG-CoA reductase rather than activating PPARα [23,37,38]. However, statins can reduce ANGPTL8 in a dose-dependent manner [39]. This potential confounding should be considered when interpreting the observed associations, especially acknowledging the sample size.

In summary, while our findings suggest a potential link between reduced ANGPTL4 levels and peripheral diabetic neuropathy, as well as metabolic disturbances in T2DM, these associations were attenuated after statistical adjustment. ANGPTL8 showed limited independent associations with cardiac autonomic neuropathy. These results add to the growing body of evidence that the ANGPTL3-4-8 axis represents a promising therapeutic target in metabolic disease. Pharmacological modulation of ANGPTL4 (e.g., through monoclonal antibodies or gene-silencing approaches) has already shown triglyceride-lowering effects in early clinical trials, while ANGPTL8 inhibition is being explored for its potential to improve postprandial lipid handling and reduce cardiovascular risk [40,41,42]. Future studies should employ longitudinal designs, larger multi-center cohorts, and tissue-specific analyses to clarify the temporal and causal relationships between ANGPTL4/8 dysregulation and diabetic complications. The integration of ANGPTL measurements with advanced imaging (e.g., corneal confocal microscopy for small-fiber neuropathy) and multi-omics approaches may further refine their utility as biomarkers for risk stratification and personalized therapy in T2DM.

## 5. Conclusions

In summary, lower circulating ANGPTL4 is associated with the presence of T2DM, peripheral diabetic neuropathy, and key metabolic derangements, whereas ANGPTL8 shows potential as an unadjusted marker of cardiac autonomic neuropathy. These findings suggest that ANGPTL4 and ANGPTL8 may reflect underlying metabolic and inflammatory disturbances in patients with diabetic neuropathy. Larger prospective studies are required to further evaluate the potential role of these proteins as biomarkers in the context of diabetic neuropathy and to better understand the contribution of the ANGPTL3-4-8 axis to the development and progression of neuropathic complications in type 2 diabetes.

## Figures and Tables

**Table 1:** Baseline characteristics of the study groups.

	Group 1 *Diabetes Mellitus*	Group 2 *Control Group*
Age (y)	58.6 ± 8.2 *	51.84 ± 7.8
Weight (kg)	98.2 ± 17.9 *	81.3 ± 19.2
BMI (kg/m^2^)	34.9 ± 5.8 *	29.9 ± 6.3
Waist circumference (cm)	112.3 ± 14.2 *	105.0 ± 10.9
Waist-to-hip ratio	0.98 ± 0.11 *	0.90 ± 0.08
Waist-to-stature ratio	0.76 ± 0.07 *	0.63 ± 0.05
Diabetes duration (years)	8.86 ± 4.3 *	-

* *p* < 0.05.

**Table 2:** Area under the curve.

Test Result Variable(s)	Area	Std. Error ^a^	Asymptotic Sig. ^b^	Asymptotic 95% Confidence Interval	
				Lower Bound	Upper Bound
ANGPTL4 ng/mL	0.540	0.064	0.538	0.414	0.667
ANGPTL8 pg/mL	0.678	0.061	0.007	0.558	0.799

The test result variable(s): ANGPTL4 ng/mL, ANGPTL8 pg/mL have at least one tie between the positive actual state group and the negative actual state group. Statistics may be biased. a. Under the nonparametric assumption. b. Null hypothesis: true area = 0.5.

**Table 3:** Association of ANGPTL4 and ANGPTL8 with various biochemical and metabolic markers.

Marker	ANGPTL4_r	ANGPTL4_p	ANGPTL4_FDR	ANGPTL8_r	ANGPTL8_p	ANGPTL8_FDR
Weight (n = 160)	−0.224	0.00435	0.02309	−0.082	0.30147	0.63643
BMR (n = 130)	−0.251	0.00457	0.02309	−0.175	0.05055	0.36587
Height (n = 160)	−0.179	0.0236	0.07472	−0.15	0.05777	0.36587
Fat (%) (n = 130)	0.173	0.05412	0.12079	0.047	0.60547	0.88491
BMI (n = 160)	−0.125	0.11401	0.21662	−0.006	0.9432	0.9432
Visceral fat rating (n = 130)	−0.12	0.18122	0.28692	−0.141	0.11548	0.39732
TBW% (n = 130)	−0.126	0.16142	0.27881	−0.022	0.80344	0.9432
Fat mass (n = 130)	0.03	0.73513	0.77597	−0.063	0.48631	0.84859
Waist circumference (n = 160)	−0.043	0.65504	0.75324	0.032	0.73974	0.937
Hip circumference (n = 160)	−0.001	0.99354	0.99354	−0.039	0.68284	0.92671
Insulin (n = 65)	−0.171	0.03165	0.08591	−0.011	0.89512	0.9432
Fasting glucose (n = 160)	0.084	0.61115	0.75324	−0.094	0.57115	0.88491
HbA1c (n = 93)	−0.044	0.67395	0.75324	0.008	0.94304	0.9432
eGFR (n = 160)	0.11	0.21333	0.31179	0.136	0.12547	0.39732
ACR (n = 93)	0.083	0.46375	0.62938	−0.141	0.20994	0.56983
GGT (n = 160)	−0.293	0.0011	0.02083	−0.101	0.2692	0.63643
ALAT (n = 160)	−0.209	0.01767	0.06716	−0.17	0.05343	0.36587
ASAT (n = 160)	−0.168	0.05722	0.12079	−0.061	0.49129	0.84859

Correlations are Spearman’s r between each marker and the log-transformed ANGPTL4 or ANGPTL8. FDR values represent *p*-values corrected using the Benjamini–Hochberg method for the set of markers shown in the table. n varies depending on the marker due to missing data.

## Data Availability

Data and models may be shared under a research agreement with any other researchers working in non-profit organizations.

## Abbreviations

ACE	Angiotensin-converting enzyme
ACR	Albumin-to-creatinine ratio
ALAT	Alanine aminotransferase
ANGPTL4	Angiopoietin-like protein 4
ANGPTL8	Angiopoietin-like protein 8
ASAT	Aspartate aminotransferase
BMI	Body mass index
BMR	Basic metabolic rate
CAN	Cardiac autonomic neuropathy
CKD	Chronic kidney disease
DAN	Diabetic autonomic neuropathy
GGT	Gamma-glutamyl transferase
NAFLD	Non-alcoholic fatty liver disease
WHR	Waist-to-hip ratio
WSR	Waist-to-stature ratio
